# Preferences and acceptability for long‐acting PrEP agents among pregnant and postpartum women with experience using daily oral PrEP in South Africa and Kenya

**DOI:** 10.1002/jia2.26088

**Published:** 2023-05-23

**Authors:** Nafisa J. Wara, Rufaro Mvududu, Mary M. Marwa, Laurén Gómez, Nyiko Mashele, Catherine Orrell, Corrina Moucheraud, John Kinuthia, Grace John‐Stewart, Landon Myer, Risa Hoffman, Jillian Pintye, Dvora L. Joseph Davey

**Affiliations:** ^1^ David Geffen School of Medicine at the University of California Los Angeles California USA; ^2^ Division of Epidemiology and Biostatistics School of Public Health University of Cape Town Cape Town South Africa; ^3^ Department of Research and Programs Kenyatta National Hospital Nairobi Kenya; ^4^ Department of Global Health University of Washington Seattle Washington USA; ^5^ Desmond Tutu HIV Centre Institute of Infectious Disease and Molecular Medicine and the Department of Medicine University of Cape Town Cape Town South Africa; ^6^ Department of Health Policy and Management Fielding School of Public Health University of California Los Angeles Los Angeles California USA; ^7^ Department of Epidemiology University of Washington Seattle Washington USA; ^8^ Department of Medicine University of Washington Seattle Washington USA; ^9^ Department of Pediatrics University of Washington Seattle Washington USA; ^10^ Division of Infectious Diseases David Geffen School of Medicine at the University of California Los Angeles California USA; ^11^ Department of Biobehavioral Nursing and Health Informatics University of Washington Seattle Washington USA; ^12^ Department of Epidemiology Fielding School of Public Health University of California Los Angeles Los Angeles California USA

**Keywords:** breastfeeding, long‐acting, PrEP, pregnancy, Kenya, South Africa

## Abstract

**Introduction:**

Long‐acting pre‐exposure prophylaxis (PrEP) options could overcome barriers to oral PrEP persistence during pregnancy and postpartum. We evaluated long‐acting PrEP preferences among oral PrEP‐experienced pregnant and postpartum women in South Africa and Kenya, countries with high PrEP coverage with pending regulatory approvals for long‐acting injectable cabotegravir and the dapivirine vaginal ring (approved in South Africa, under review in Kenya).

**Methods:**

From September 2021 to February 2022, we surveyed pregnant and postpartum women enrolled in oral PrEP studies in South Africa and Kenya. We evaluated oral PrEP attitudes and preferences for long‐acting PrEP methods in multivariable logistic regression models adjusting for maternal age and country.

**Results:**

We surveyed 190 women in South Africa (67% postpartum; median age 27 years [IQR = 22–32]) and 204 women in Kenya (79% postpartum; median age 29 years [IQR = 25–33]). Seventy‐five percent of participants reported oral PrEP use within the last 30 days. Overall, forty‐nine percent of participants reported negative oral PrEP attributes, including side effects (21% South Africa, 30% Kenya) and pill burden (20% South Africa, 25% Kenya). Preferred PrEP attributes included long‐acting method, effectiveness, safety while pregnant and breastfeeding, and free medication. Most participants (75%, South Africa and Kenya) preferred a potential long‐acting injectable over oral PrEP, most frequently for a longer duration of effectiveness in South Africa (87% South Africa, 42% Kenya) versus discretion in Kenya (5% South Africa, 49% Kenya). Eighty‐seven percent of participants preferred oral PrEP over a potential long‐acting vaginal ring, mostly due to concern about possible discomfort with vaginal insertion (82% South Africa, 48% Kenya). Significant predictors of long‐acting PrEP preference included past use of injectable contraceptive (aOR = 2.48, 95% CI: 1.34, 4.57), disliking at least one oral PrEP attribute (aOR = 1.72, 95% CI: 1.05, 2.80) and preferring infrequent PrEP use (aOR = 1.58, 95% CI: 0.94, 2.65).

**Conclusions:**

Oral PrEP‐experienced pregnant and postpartum women expressed a theoretical preference for long‐acting injectable PrEP over other modalities, demonstrating potential acceptability among a key population who must be at the forefront of injectable PrEP rollout. Reasons for PrEP preferences differed by country, emphasizing the importance of increasing context‐specific options and choice of PrEP modalities for pregnant and postpartum women.

## 1 INTRODUCTION

HIV incidence remains high among cisgender women of reproductive age in South Africa and Kenya, including during pregnancy and postpartum [1, 2, 3, 4]. Pregnant women without HIV are at elevated risk of HIV acquisition due to structural and socio‐cultural factors (e.g. poverty and gender inequity) that may result in high‐risk scenarios, including not knowing the HIV status of partner(s), engaging in condomless sex and having multiple sex partners [5, 6, 7, 8]. Furthermore, the risk of vertical HIV transmission is elevated during pregnancy and breastfeeding due to acute maternal HIV infection [2, 3].

Daily oral tenofovir disoproxil fumarate/emtricitabine (TDF/FTC) pre‐exposure prophylaxis (PrEP) for HIV prevention is being rapidly scaled in South Africa and Kenya, and these two countries have the highest and second highest number of PrEP initiations globally [9]. Studies have reported high rates of oral PrEP uptake among pregnant and postpartum women in South Africa and Kenya [10, 11]. However, the delivery of oral PrEP among pregnant and postpartum women is challenged by insufficient integration of PrEP provision and counselling into existing antenatal and postpartum healthcare [12]. Furthermore, self‐reported and objective levels of adherence on oral PrEP were low among pregnant and postpartum women in Kenya and South Africa [11, 13]. Substantial barriers to effective PrEP use include pill burden, stigma and limited disclosure of PrEP, and financial and logistical barriers to accessing a clinic for PrEP [14, 15, 16]. Strategies to overcome barriers to effective PrEP use are urgently needed.

Long‐acting modalities, such as injectable cabotegravir (CAB‐LA) and the dapivirine vaginal ring (DVR), may improve PrEP initiation and persistence among pregnant and postpartum women. CAB‐LA, an intramuscular injection administered every 8 weeks, is superior to oral PrEP, reducing the risk of HIV infection by 88% compared to daily oral TDF/FTC among cisgender women in sub‐Saharan Africa [17]. Preliminary data show that CAB‐LA is well tolerated during pregnancy and has a similar pharmacokinetic profile to its use by non‐pregnant women [18, 19]. CAB‐LA received regulatory approval in the United States in December 2021, in South Africa in December 2022, and is pending approval in Kenya and other countries in East and Southern Africa [9]. The DVR, inserted vaginally and replaced every 4 weeks, has been shown to reduce the risk of HIV infection by approximately 30% and is not associated with adverse pregnancy or infant outcomes [20]. The DVR received regulatory approval in South Africa in March 2022 (not yet approved among pregnant women), and is pending approval in Kenya [9]. These developments highlight the urgent need to understand potential facilitators and barriers to the uptake and persistence of these novel PrEP methods among pregnant and postpartum women. Our study aims to assess preferences for and perceptions of long‐acting methods among oral PrEP‐experienced pregnant and postpartum women in Kenya and South Africa.

## 2 METHODS

### 2.1 Study participants

We conducted surveys with participants enrolled in an observational cohort study assessing daily oral PrEP initiation and persistence among pregnant and breastfeeding women (PBFW) in Cape Town, South Africa (PrEP‐PP) and an observational extension cohort of a cluster randomized trial offering daily oral PrEP among PBFW in Western Kenya (PrIMA‐X) [21, 22]. Eligibility criteria for PrEP‐PP included: ≥16 years old, confirmed HIV‐negative serostatus by a fourth‐generation antigen/antibody combination HIV test (Abbott), intention to stay in Cape Town through the postpartum period and no contraindications to PrEP use. Eligibility criteria for PrIMA‐X included: ≥15 years old, confirmed HIV and TB negative, intention to reside in the area for at least 1 year postpartum, and plans to receive postnatal and infant care at the study facility. Enrolment criteria for this sub‐sample included: currently using or having previously used daily oral PrEP; currently pregnant or postpartum; and enrolment in PrEP‐PP or PrIMA‐X.

In PrEP‐PP, participants were offered oral PrEP at enrolment and received a 3‐month prescription to correspond with quarterly study visits until birth or the first postpartum visit. In PrIMA‐X, participants were offered oral PrEP at enrolment and returned for study visits that aligned with country antenatal care guidelines (monthly during pregnancy and at intervals of weeks to months postpartum). Participants in both studies received PrEP counselling and completed surveys at follow‐up visits, including questions on PrEP use and adherence through self‐report and pill‐count measures as well as dried blood spots of tenofovir‐diphosphate (levels not shared with participants) [21, 22].

### 2.2 Data collection

Between September 2021 and February 2022, trained study staff fluent in English and either isiXhosa, Kiswahili or Luo approached women attending PrEP‐PP or PrIMA‐X follow‐up visits to introduce the study. Study staff then screened participants for study eligibility, obtained written informed consent in English or the participant's local language (isiXhosa, Kiswahili or Luo) and administered the survey to eligible consenting participants. Study staff asked participants survey questions and recorded responses on a tablet. The survey took 30–40 minutes and was completed on REDCap, a secure web‐based platform [23]. Participants received 120 Rand in South Africa (∼$7 USD) or KSh 300 in Kenya (∼$3 USD) for their participation in the study as well as transportation expenses.

#### 2.2.1 Survey measures

##### 2.2.1.1 Participant socio‐demographic characteristics

We collected (1) basic demographic data (including age, obstetric history, education level, employment and number of sexual partners); (2) HIV risk perception; (3) current alcohol use adapted from the Alcohol Use Disorders Identification Test [24, 25]; (4) PrEP adherence based on 30‐day recall (i.e. responding “yes” or “no” to taking PrEP within the past 30 days); (5) clinic access (including transportation method, total travel time and total transportation cost); and (6) previous contraceptive method use. We also assessed experienced or perceived PrEP stigma using a 7‐item scale derived from existing literature in which participants responded to statements describing experiences of PrEP stigma [26, 27].

##### 2.2.1.2 Current and future PrEP preferences

We assessed perceptions of daily oral PrEP by asking participants what they like and dislike about oral PrEP. We described methods of long‐acting HIV prevention currently pending regulatory approval or in development, and asked participants about preferences regarding HIV prevention modalities that may be available in the future. We adapted a list of PrEP characteristics from a discrete choice experiment of HIV prevention methods assessed within the Quatro Study [28]. We asked participants to rank the top three most important characteristics of a potential HIV prevention product, the top three most important access‐related characteristics and how frequently they would theoretically use an HIV prevention method.

##### 2.2.1.3 Long‐acting PrEP preferences

We provided information regarding CAB‐LA and the DVR (Table S1) and assessed preferences regarding injectable PrEP and the DVR by asking participants whether they would prefer to switch to the long‐acting method or remain on oral PrEP. We then assessed reasons for preferring the long‐acting method or oral PrEP.

### 2.3 Statistical analyses

We used descriptive statistics (median, interquartile range [IQR] and frequency) to report participant responses, and used Chi‐square, Fischer's Exact and Wilcoxon rank sum to compare responses between countries. We used univariate and multivariable logistic regression models to assess predictors of preferring a long‐acting PrEP method (injection or ring) over oral PrEP among those with a preference for long‐acting PrEP. Predictors considered statistically significant in univariate logistic regression models were included in multivariable logistic regression models, adjusting for maternal age and country as *a priori* potential confounders. We used two‐tailed tests to evaluate the significance of regression models, with a significance threshold of *p*<0.10. Statistical analyses were conducted with STATA v.17 [29].

### 2.4 Ethics

The PrEP‐PP study was approved by the Human Research Ethics Committee of the University of Cape Town Faculty of Health Sciences (#297/2018) and by the University of California, Los Angeles Institutional Review Board (IRB#18‐001622). The PrIMA‐X study was approved by the Kenyatta National Hospital‐University of Nairobi Ethics and Research Committee (P73/02/2017) and by the University of Washington Human Subjects Division (STUDY00000438).

## 3 RESULTS

Overall, 394 women were enrolled in the study, 190 women (out of 220 eligible participants approached; 86%) from the South African study (*N* = 1201) and 204 (none declined, 100%) from the Kenyan study (*N* = 1300). Median ages were 27 (IQR = 22–32) and 29 (IQR = 25–33), respectively (Table 1). Overall, 33% of South African participants were pregnant (*n* = 63), while 21% of Kenyan participants were pregnant (*n* = 42), with the remaining in both groups postpartum. Most participants completed some or all secondary school education (South Africa 93%, Kenya 74%, *p*<0.01) and were unemployed (South Africa 72%, Kenya 87%, *p*<0.01). Almost all women reported having ≥1 current sexual partner (South Africa 92%, Kenya 97%, *p* = 0.03). Most participants reported that they took at least one dose of PrEP over the previous 30 days at the time of the survey (South Africa 82%, Kenya 68%, *p*<0.01), and the median time on PrEP among current PrEP users was 337 days (IQR = 263–420) and 308 days (IQR = 114–442) among South African and Kenyan participants, respectively.

**Table 1:** Demographics and health characteristics of pregnant and postpartum women with experience taking oral PrEP, South Africa and Kenya, September 2021–February 2022 (*N* = 394 women).

	Overall (*N* = 394, %)	South Africa (*n* = 190, %)	Kenya (*n* = 204, %)	*p*‐value
Age (median, IQR)	28 [24–32]	27 [22–32]	29 [25–33]	<0.01
Pregnant (n, %)	105 (27)	63 (33)	42 (21)	0.01
Postpartum (n, %)	289 (73)	127 (67)	162 (79)	0.01
Days on oral PrEP (median, IQR)	335 [168–420]	337 [263–420]	308 [114–442]	0.04
Last grade completed (n, %)				
Primary school (Grades 1–6)	28 (7)	2 (1)	26 (13)	<0.01
Some secondary school (Grades 7–11)	236 (60)	106 (56)	130 (64)	
Completed secondary school	90 (23)	70 (37)	20 (9)	
Some or all tertiary	40 (10)	12 (6)	28 (14)	
Currently employed (formally or informally)? (n, %)				
No	313 (79)	136 (72)	177 (87)	<0.01
Yes	80 (20)	54 (28)	26 (13)	
Prefer not to answer	1 (1)	0 (0)	1 (1)	
Currently have at least one sexual partner (n, %)				
No	21 (5)	15 (8)	6 (3)	0.03
Yes	373 (95)	175 (92)	198 (97)	
HIV risk perception^a^ (n, %)				
No risk at all	121/380 (32)	84/176 (48)	37 (18)	<0.01
Small chance	145/380 (44)	56/176 (32)	89 (44)	
Moderate chance	89/380 (34)	20/176 (11)	69 (34)	
Great chance	23/380 (6)	16/176 (9)	7 (3)	
Prefer not to answer	2/380 (1)	0/176 (0)	2 (1)	
Current alcohol use (n, %)				
No	363/380 (95)	164/176 (92)	199 (98)	0.31
Yes^b^	17/380 (5)	12/176 (8)	5 (3)	
HIV PrEP Stigma^c^ (n, %)				
Endorsed 0 forms of stigma	251/380 (66)	128/176 (73)	123 (60)	0.01
Endorsed >1 form of stigma	129/380 (34)	48/176 (27)	81 (40)	
PrEP adherence over the past 30 days (n, %)				
No	100 (25)	34 (18)	66 (32)	<0.01
Yes	294 (75)	156 (82)	138 (68)	
Clinic transportation (n, %)				
Walking	31 (8)	16 (8)	15 (7)	<0.01
Taxi/Minibus	206 (52)	172 (91)	34 (17)	
Motorbike	152 (38)	0 (0)	152 (75)	
Other	5 (2)	2 (1)	3 (1)	
Total travel time to clinic and return (minutes, median, IQR)	40 [30–60]	30 [20.0–40.0]	55 [31.0–60.0]	<0.01
Cost to travel to clinic and return (USD, median, IQR)	1.32 [1.31–1.74]	1.32 [1.32–1.32]	1.75 [0.88–2.64]	0.02
Have you ever used any of the following family planning methods? (n, %)				
Male condom	217 (55)	171 (90)	46 (23)	<0.01
Oral contraceptive pill	98 (25)	42 (22)	56 (27)	0.22
Injectable contraceptive	312 (79)	178 (94)	134 (66)	<0.01
Contraceptive implant	181 (46)	70 (37)	111 (54)	<0.01
Other (female condom, vaginal ring, IUD/loop, tubal ligation)	18 (5)	9 (5)	9 (4)	0.27
None	11 (3)	1 (1)	9 (4)	0.01

^a^ Participants were asked “How would you describe your chances of getting HIV in the next year?” with the option to select “No risk at all,” “Small chance,” “Moderate chance,” “Great chance” or “Prefer not to answer.”

^b^ Combined categories: “Once a month or less, 2–4 times a month, 2–3 times a week, 4 or more times a week.”

^c^ Participants were asked to respond on a 5‐point Likert scale from “Strongly Disagree” to “Strongly Agree” to 7 statements on potential stigma experienced regarding PrEP use (i.e. “I feel ashamed of using PrEP,” “I feel embarrassed about using PrEP”).

At entry into the parent study, 52% of South African women and 81% of Kenyan women reported any perceived risk of HIV acquisition (*p*<0.01). More than half endorsed no forms of PrEP stigma (66%, *n* = 251/380), although more women in Kenya endorsed at least one form of PrEP stigma (Kenya 40%, South Africa 27%, *p* = 0.01). When asked about any previous experience with contraceptives, women most frequently described the use of injectable contraceptives (South Africa 94% vs. Kenya 66%, *p*<0.01). Injectable contraceptives were followed in South Africa by use of male condoms (*n* = 171, 90%) and contraceptive implants (*n* = 70, 37%), and in Kenya by contraceptive implants (*n* = 111, 54%) and male condoms (*n* = 46, 23%).

### 3.1 Experiences with oral PrEP

Almost all participants (*n* = 386, 98%) reported efficacy in HIV prevention as a positive characteristic of daily oral PrEP, followed by daily oral PrEP having few or no side effects (*n* = 61, 15%) and being easy to use (*n* = 27, 7%) (Table 2). Forty‐two percent of women in South Africa (*n* = 79) and fifty‐six percent in Kenya (*n* = 114, *p*<0.01) disliked at least one attribute about daily oral PrEP. The most frequently reported dislikes included side effects (South Africa 21%, Kenya 30%, *p* = 0.03), daily use (South Africa 20%, Kenya 25%, *p* = 0.20) and taking it orally (South Africa 6%, Kenya 11%, *p* = 0.08).

**Table 2:** Likes and dislikes regarding daily oral PrEP in pregnant and postpartum women.

Oral PrEP likes^a^	Overall (*N* = 394)	South Africa (*n* = 190)	Kenya (*n* = 204)	*p*‐value
HIV prevention	386 (98)	186 (98)	200 (98)	0.92
PrEP has few/no side effects	61 (15)	35 (18)	26 (13)	0.12
Ease of use	27 (7)	16 (8)	11 (5)	0.23
No interruption of sex (as is needed for condoms)	16 (4)	5 (3)	11 (5)	0.17
Easy to hide	10 (3)	0 (0)	10 (5)	<0.01
Take it daily	8 (2)	5 (3)	3 (2)	0.88
Taken orally	7 (2)	5 (3)	2 (1)	0.95
PrEP is safe for the baby	5 (1)	5 (3)	0 (0)	0.03
Other (e.g. overall safety, increased appetite, peaceful sleep)	4 (1)	4 (2)	0 (0)	0.05
Nothing	1 (1)	0 (0)	1 (1)	0.52

Oral PrEP dislikes^a^	Overall (*N* = 394)	South Africa (*n* = 190)	Kenya (*n* = 204)	*p*‐value
Side effects	100 (25)	39 (21)	61 (30)	0.03
Must take it daily	90 (23)	38 (20)	52 (25)	0.20
Must take orally	35 (9)	12 (6)	23 (11)	0.08
Pill size is too big	12 (3)	9 (5)	3 (2)	0.99
No STI prevention	10 (3)	4 (2)	6 (3)	0.42
Not discreet	9 (2)	0 (0)	9 (4)	<0.01
Other (e.g. general dislike, pill taste, pill smell)	3 (1)	2 (1)	1 (1)	0.90
Nothing	201 (51)	111 (58)	90 (44)	<0.01

^a^ Participants were asked open‐ended questions regarding oral PrEP likes/dislikes and interviewer selected all responses endorsed by the participant.

### 3.2 Future PrEP preferences

When asked to rank characteristics of a potential PrEP product that may be available to them in the future, 52% of participants (*n* = 203) ranked effectiveness at preventing HIV as the most important, followed by the ability to have a healthy pregnancy (*n* = 47, 12%), frequency of use (*n* = 39, 10%), tolerability (*n* = 39, 10%) and the ability to breastfeed and have a healthy baby (*n* = 26, 7%). However, there were country‐specific differences in the ranking of several attributes: more women in Kenya ranked HIV prevention highest (78%, South Africa 23%, *p*<0.01), and more women in South Africa ranked having a healthy pregnancy (19%, Kenya 5%, *p*<0.01), frequency of use (18%, Kenya 2%, *p*<0.01), side effects (16%, Kenya 4%, *p*<0.01) and privacy (6%, Kenya 1%, *p* = 0.01) as most important (Table 3). When asked to rank access‐related characteristics of a potential PrEP product, 52% of participants ranked medication being free as the most important, followed by ease of the process by which the product is obtained, location and total time it takes to get the product. When asked to rank preferred frequency of PrEP use, participants most frequently preferred once a year (*n* = 123, 31%), followed by once per month (*n* = 62, 16%), once every 2–3 months (*n* = 59, 15%), before sex (*n* = 53, 13%), every day (*n* = 46, 12%) and once every 6 months (*n* = 45, 11%).

**Table 3:** Ranked characteristics of potential HIV prevention products among pregnant and postpartum women.

Most important characteristic of a potential HIV prevention product (ranked #1)	Overall (*N* = 394)	South Africa (*n* = 190)	Kenya (*n* = 204)	*p*‐value
Effective at preventing HIV	203 (52)	44 (23)	159 (78)	<0.01
Healthy pregnancy	47 (12)	36 (19)	11 (5)	<0.01
How frequently it is used (e.g. before sex, once a day, once a month)	39 (10)	35 (18)	4 (2)	<0.01
Side effects	39 (10)	31 (16)	8 (4)	<0.01
Ability to breastfeed and have healthy baby	26 (7)	15 (8)	11 (5)	0.32
How it is used (e.g. vaginal ring, injected, pill)	25 (6)	16 (8)	9 (4)	0.10
Privacy (from partner)	13 (3)	11 (6)	2 (1)	0.01
Other (not forgotten easily, overall safety)	2 (1)	2 (1)	0 (0)	0.23

Most important access‐related characteristic of a potential HIV prevention product (ranked #1)				
Free of charge	203 (52)	104 (55)	99 (49)	0.22
How easy it is to obtain	79 (20)	43 (23)	36 (18)	0.22
Where to pick up (clinic, pharmacy, community)	48 (12)	28 (15)	20 (10)	0.14
Total time it takes to get product	31 (8)	15 (8)	16 (8)	0.99

Most preferable frequency of HIV prevention method (ranked #1)				
Before sex	53 (13)	30 (16)	23 (11)	0.19
After sex	6 (2)	5 (3)	1 (1)	0.10
Every day	46 (12)	26 (14)	20 (10)	0.23
Once per month	62 (16)	25 (13)	37 (18)	0.18
Once every 2–3 months	59 (15)	17 (9)	42 (21)	<0.01
Once every 6 months	45 (11)	11 (6)	34 (17)	<0.01
Once yearly	123 (31)	76 (40)	47 (23)	<0.01

### 3.3 Preference of long‐acting PrEP modalities

**Figure 1:**
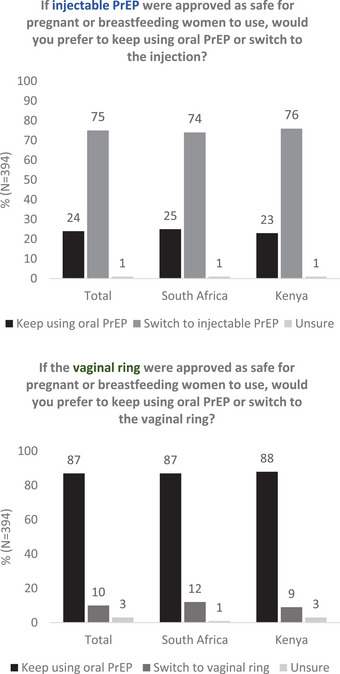
Acceptability of long‐acting PrEP methods versus daily oral PrEP by pregnant and postpartum women in Kenya and South Africa (*n* = 394 women). Participants (*N* = 394) were asked the following questions: “If injectable PrEP were approved as safe for pregnant or breastfeeding women to use, would you prefer to keep using oral PrEP or switch to the injection?” and “If the vaginal ring were approved as safe for pregnant or breastfeeding women to use, would you prefer to keep using oral PrEP or switch to the vaginal ring?” Participants either expressed interest in continuing to use oral PrEP, switching to the long‐acting method or were unsure, as represented by the dark black, medium grey and light grey bars, respectively. The left axis shows frequency of responses in percent. Responses are presented by country as well as combined (“Total”).

Overall, three‐fourths of participants (*n* = 297, South Africa 74%, Kenya 76%, *p* = 0.60) responded that they would prefer to switch to injectable PrEP over remaining on oral PrEP if it were available (Figure 1). South African and Kenyan women differed in their reasons for preferring injectable PrEP over oral PrEP: participants in South Africa more commonly preferred it for its longer duration of effectiveness (South Africa 87%, Kenya 42%, *p*<0.01) and not having to take PrEP daily (South Africa 57%, Kenya 41%, *p*<0.01), while participants in Kenya were more likely to state reasons of privacy (Kenya 49%, South Africa 5%, *p*<0.01) and not having to carry pills (Kenya 42%, South Africa 13%, *p*<0.01) (Table S2). The most common concerns regarding injectable PrEP were injection pain and potential side effects. Although overall these concerns were not frequently reported, Kenyan participants were more concerned than South African participants regarding the safety of the new injectable (Kenya 13%, South Africa 4%, *p* = 0.01) and its safety for infants if using the injectable while breastfeeding (Kenya 6%, South Africa 0%, *p* = 0.01).

Fewer women (*n* = 40, 10%) would prefer switching to the DVR over oral PrEP in both South Africa and Kenya (12% and 9%, respectively). South African women were interested in the DVR most frequently due to its longer duration (*n* = 14/22, 64%) and not having to remember to take a daily pill (*n* = 12/22, 55%), while most common reasons for preferring the DVR in Kenya were because it was perceived to be “easy to use” (*n* = 8/18, 44%) or did not involve carrying pills (*n* = 6/18, 33%). Participants were unsure or preferred oral PrEP over the DVR most frequently due to its insertion into the vagina particularly among South African women (South Africa 82%, Kenya 48%, *p*<0.01), potential side effects more so among Kenyan women (South Africa 21%, Kenya 33%, *p* = 0.02) and concerns about safety in both sub‐groups (South Africa 23%, Kenya 27%). Participants in South Africa were also more frequently concerned about the DVR not providing sufficient protection against HIV (14%, Kenya 7%, *p* = 0.03).

In multivariable analyses adjusting for age and country, preference of a long‐acting PrEP method (either injectable PrEP or DVR) over daily oral PrEP was associated with prior use of injectable contraception (aOR = 2.48, 95% CI = 1.34, 4.57), disliking at least one attribute of daily oral PrEP (aOR = 1.72, 95% CI = 1.05, 2.80), disliking the daily use of oral PrEP (aOR = 1.92, 95% CI = 1.01, 3.67) and preferring longer duration of effectiveness (aOR = 1.58, 95% CI = 0.94, 2.65) (Table 4).

**Table 4:** Factors associated with long‐acting PrEP preference versus oral PrEP among PrEP‐experienced pregnant and postpartum women, *N* = 394.

	Summary statistics					
	Wants long‐acting^a^ (*n* = 305, %)	Wants oral PrEP (*n* = 89, %)	Unadjusted OR (95% CI)	*p*‐value	Adjusted OR^b^ (95% CI)	*p*‐value
*Maternal age (median, IQR) years*	28 [24‐32]	27 [23‐31]	1.03 [0.99‐1.08]	0.11	1.04 [0.99, 1.08]	0.11
≥25 years	221 (72)	59 (66)	1.34 [0.81‐2.22]	0.26	1.35 [0.81‐2.27]	0.25
<25 years	84 (28)	30 (34)				
*Country*						
Kenya	158 (52)	46 (52)	1.01 [0.63‐1.61]	0.98	0.93 [0.57, 1.51]	0.77
South Africa	147 (48)	43 (48)				
*Pregnancy status*						
Pregnant	78 (26)	27 (30)	0.79 [0.47‐1.33]	0.37	0.82 [0.48, 1.39]	0.46
Postpartum	227 (74)	62 (70)				
*Time on PrEP (median, IQR) days*	331 [168‐422]	336 [185‐420]	1.00 [1.00‐1.00]	0.90	1.00 [1.00‐1.00]	0.78
*Last grade completed (ref: primary school)*	27 (9)	10 (11)	*ref*	*ref*	*ref*	*ref*
Some secondary	178 (58)	64 (72)	1.20 [0.55‐2.64]	0.64	1.12 [0.52, 2.74]	0.67
Completed secondary	72 (24)	17 (19)	1.48 [0.61‐3.61]	0.39	1.62 [0.61, 4.32]	0.34
Some or all tertiary	20 (7)	3 (3)	1.78 [0.53‐5.94]	0.35	1.68 [0.49, 5.79]	0.41
*Currently employed (formally or informally)*						
Employed	61/304 (20)	19 (21)	0.93 [0.52‐1.65]	0.79	0.81 [0.44‐1.49]	0.49
Not employed	243/304 (80)	70 (79)				
*At least one sexual partner*						
≥1 sexual partner	288 (94)	85 (96)	0.80 [0.26‐2.43]	0.69	0.75 [0.24‐2.30]	0.61
0 sexual partners	16 (5)	4 (4)				
*PrEP stigma (median, IQR)*	14 [13‐17]	15 [13‐16]	1.02 [0.96‐1.09]	0.56	1.03 [0.96‐1.10]	0.45
Endorsed 0 stigma	193/293 (66)	58/87 (67)	1.03 [0.62‐1.72]	0.89	1.06 [0.63‐1.77]	0.83
Endorsed ≥1 stigma statement	100/293 (34)	29/87 (33)				
*HIV Risk Perception*						
Any perceived HIV risk	194/291 (67)	63/87 (72)	0.76 [0.45, 1.29]	0.31	0.75 [0.43, 1.31]	0.31
No perceived HIV risk	97/291 (33)	24/87 (28)				
*Alcohol use*						
Endorses any alcohol use	12/293 (4)	5/87 (6)	0.70 [0.24‐2.05]	0.52	0.73 [0.25‐2.17]	0.58
Does not endorse alcohol use	281/293 (96)	82/87 (94)				
*PrEP persistence (has the participant taken PrEP over the past 30 days?)*						
Yes	222 (73)	72 (81)	0.63 [0.35‐1.13]	0.12	0.65 [0.36, 1.19]	0.16
No	83 (27)	17 (19)				
*Time traveled to clinic and return (median, IQR) minutes*	40 [30‐60]	40 [30‐60]	1.00 [0.99‐1.01]	0.95	0.99 [0.99‐1.01]	0.99
*Cost to travel to clinic and return (median, IQR) USD equivalent*	1.29 [1.29‐1.71]	1.29 [1.29‐1.71]	1.12 [0.86‐1.45]	0.40	1.11 [0.85‐1.45]	0.44
*Contraceptive used in the past*						
Injection	**252 (83)**	**60 (67)**	**2.30 [1.35‐3.92]**	**0.002**	**2.48 [1.34, 4.57]**	**<0.01**
Condom	172 (56)	45 (51)	1.26 [0.79‐2.03]	0.33	1.59 [0.82, 3.07]	0.17
Implant	134 (44)	47 (53)	0.70 [0.44‐1.12]	0.14	0.71 [0.44, 1.16]	0.17
Oral contraceptive	79 (26)	19 (21)	1.29 [0.73‐2.27]	0.38	1.22 [0.69‐2.17]	0.50
*Oral PrEP likes*			–	–	—	—
HIV prevention	298 (98)	87 (98)	0.98 [0.20‐4.80]	0.98	0.95 [0.19‐4.66]	0.95
No side effects	50 (16)	11 (12)	1.39 [0.69‐2.80]	0.36	1.43 [0.71‐2.90]	0.32
Ease of use	21 (7)	6 (7)	1.02 [0.40‐2.62]	0.96	1.07 [0.41‐2.75]	0.90
No interruption of sex	12 (4)	4 (4)	0.87 [0.27‐2.77]	0.81	0.87 [0.27‐2.80]	0.82
Easy to hide	7 (2)	3 (3)	0.67 [0.17‐2.66]	0.57	0.63 [0.16‐2.56]	0.52
Take it daily	**4 (1)**	**4 (4)**	**0.28 [0.07‐1.15]**	**0.08**	**0.30 [0.07‐1.24]**	**0.10**
Take it orally	4 (1)	3 (3)	0.38 [0.08‐1.73]	0.21	0.38 [0.08‐1.77]	0.22
Nothing	1 (0.3)	0 (0)				
*Oral PrEP dislikes*						
Dislikes at least one thing about oral PrEP	**158 (52)**	**35 (39)**	**1.66 [1.03‐2.68]**	**0.04**	**1.72 [1.05, 2.80]**	**0.03**
Side effects	81 (27)	18 (20)	1.43 [0.80‐2.54]	0.23	1.43 [0.80, 2.55]	0.23
Daily use	**75 (25)**	**13 (15)**	**1.91 [1.00‐3.63]**	**0.05**	**1.92 [1.01, 3.67]**	**0.05**
Take it orally	28 (9)	7 (8)	1.18 [0.50‐2.81]	0.70	1.20 [0.50, 2.85]	0.69
Not discreet	7 (2)	2 (2)	1.02 [0.21‐5.01]	0.98	1.09 [0.22, 5.46]	0.92
No STI protection	6 (2)	4 (4)	0.43 [0.12‐1.55]	0.20	0.45 [0.12, 1.64]	0.23
*Preferred frequency of PrEP use*						
Ranked every month, 2‐3 months, 6 months, or year #1	**230 (75)**	**59 (66)**	**1.56 [0.94‐2.60]**	**0.09**	**1.58 [0.94, 2.65]**	**0.08**
Ranked before sex, after sex, or every day #1	75 (25)	30 (34)				
*Interested in community PrEP delivery*						
Yes	131 (43)	32 (36)	1.34 [0.82‐2.19]	0.24	1.45 [0.85, 2.45]	0.17
No	174 (57)	57 (64)				

^a^ Of *n* = 305, 265 (87%) preferred the injection only over oral PrEP, 8 (3%) preferred the ring only over oral PrEP and 32 (10%) preferred both long‐acting methods over oral PrEP.

^b^ Each individual model adjusted for maternal age, country.

Bold *p*<0.10.

## 4 DISCUSSION

We identified a strong theoretical preference for long‐acting injectable PrEP among pregnant and postpartum women in Kenya and South Africa. Additionally, about one‐half of women reported liking daily oral PrEP, indicating the importance of providing pregnant and postpartum women with choices of modalities. Studies assessing end‐user preference among African women of potential HIV prevention methods (multiple vaginally inserted methods, injection and pill) showed that women had varied preference for these HIV prevention modalities, and women described the importance of personal preference in product choice [30, 31, 32]. Similar to HIV prevention, studies on contraceptive choice indicate that the best method for an individual depends on their preferences, necessitating a diversity of contraceptive options [33, 34, 35]. Our data suggest that the availability of choices to meet personal preferences is essential to improving the overall acceptability of HIV prevention methods among pregnant and postpartum women as well. Furthermore, women in South Africa and Kenya did not differ in preferences for long‐acting PrEP modalities compared to oral PrEP, but did differ in the reasoning behind their preferences, indicating the importance of context‐specific implementation when providing HIV prevention modalities. Socio‐demographic characteristics (e.g. rural vs. urban setting and community size), existing infrastructures of PrEP provision and available PrEP modalities differ between countries. Further, the prevalence of HIV and HIV treatment is significantly higher in South Africa which may impact anticipated PrEP stigma [36, 37]. These existing factors and potential contributors to differences between countries in PrEP preferences among pregnant and postpartum women need to be studied and incorporated into country‐specific plans for PrEP rollout.

Our findings on preference for long‐acting injectable PrEP among pregnant and postpartum women, as well as most reasons for their PrEP preferences, align with the existing literature among non‐pregnant women. Women also noted the need for safety data in pregnancy and lactation in our study, which was distinct for this population. In studies providing hypothetical choices between long‐acting PrEP modalities, non‐pregnant women from Kenya and South Africa preferred injectable methods of HIV prevention over other modalities, despite similar concerns regarding injection pain [32, 38, 39]. In studies assessing the acceptability of long‐acting injectable PrEP among women within clinical trials in Africa and the United States, participants described acceptability of long‐acting injectable PrEP due to ease of use and long‐term protection and would use the method again [40, 41, 42], which may suggest that hypothetical acceptability of long‐acting injectable PrEP among pregnant and postpartum women may align with its acceptability among non‐pregnant women during implementation. Previous data have identified the positive impacts of counselling about infant safety on oral PrEP initiation among women in Kenya, highlighting that obtaining further data on pregnancy outcomes and infant safety of CAB‐LA, as well as counselling on safety, may be beneficial for uptake [16].

Pregnant and postpartum women in our study reported that they would prefer to use a long‐acting injectable PrEP for privacy and discreetness of this method, highlighting the role of long‐acting PrEP to mitigate socio‐cultural barriers previously identified with oral PrEP use [14, 15, 16, 43]. Many participants in our study endorsed perceived HIV risk and PrEP stigma, which aligns with barriers to PrEP use described by pregnant and postpartum women in South Africa and Kenya in the existing literature [39, 44, 45, 46, 47, 48, 49]. Qualitative studies have described the negative community perception surrounding PrEP use experienced by pregnant and non‐pregnant women, particularly due to its association with high‐risk sexual behaviour as well as being conflated with antiretroviral therapy, leading to concealment of product use [44, 45, 46]. A qualitative study assessing oral PrEP perceptions in Kenya additionally described fear of male partners becoming violent if discovering PrEP use, indicating that stigma and negative sentiments towards PrEP within a household may decrease PrEP disclosure and necessitate concealment [47, 48]. The availability of an HIV prevention method, such as long‐acting injectable PrEP, that does not require daily administration or concealment of pills may make it easier for pregnant and postpartum women experiencing PrEP stigma to persist on the HIV prevention method—and has been hypothetically posed by women as a potential solution in previous qualitative work [46]. Long‐acting PrEP modalities must be made available in conjunction with other solutions that mitigate community‐ and individual‐level stigma experienced by women in Kenya and South Africa, such as community‐facing interventions involving media and educational initiatives, as well as the involvement of male partners in HIV prevention and education [40, 49]. Further work is necessary to assess the feasibility of incorporating long‐acting modalities into existing antenatal PrEP provision at the health facility level in South Africa and Kenya that prioritizes identified preferences of pregnant and postpartum women, such as services being free of charge or being accessible in postpartum care.

Few pregnant and postpartum women reported a preference for the DVR over oral PrEP, which may be due to unfamiliarity with the method as well as its lower efficacy. Existing acceptability and demonstration studies of the DVR show that despite similar initial concerns regarding insertion into the vagina and potential side effects, women in sub‐Saharan Africa who began using the DVR developed familiarity with the method, found it easy to integrate into their lives and reported willingness to use the method in the future [31, 50, 51, 52]. Furthermore, additional safety and efficacy data specific to the use of DVR for HIV prevention during pregnancy and lactation continue to be collated, which may further impact the future acceptability of this method among this population [53].

Prior use of injectable contraception was associated with a preference for injectable PrEP, indicating that contraceptive knowledge and familiarity with the concept of regular injections as a prevention measure may impact the uptake of injectable PrEP among pregnant and postpartum women. Previous use of contraception as a demographic characteristic in our study was representative of contraceptive use among women in African countries described in existing literature [54]. Studies assessing the acceptability of multi‐purpose prevention technologies combining HIV and pregnancy prevention similarly showed that injections were preferred compared to a ring or pill‐form of combination prevention, and that past experience with similar contraceptive delivery forms was a significant predictor of their method choice [42, 55, 56]. Combining PrEP access with family planning education may improve the uptake of HIV prevention, due to existing familiarity with long‐acting contraceptive modalities.

### 4.1 Limitations

Our study included pregnant and postpartum women with knowledge and experience with oral PrEP. As such, our results may differ from preferences of pregnant and postpartum women without prior experience using PrEP. Our surveys provided a hypothetical choice between PrEP modalities, as some methods are not yet available in South Africa and Kenya. Responses may differ from PrEP choices during an implementation offering multiple modalities. Our results may not be generalizable to other settings beyond urban South Africa and Kenya, so further study is necessary on the acceptability of long‐acting PrEP among pregnant and postpartum women in other settings. However, these findings may inform the future development and tailoring of acceptability studies assessing choice in PrEP modalities in other settings where pregnant and postpartum women experience similar barriers to PrEP use [57].

## 5 CONCLUSIONS

Our study demonstrates that pregnant and postpartum women on oral PrEP may desire to switch to long‐acting PrEP as it becomes available in South Africa and Kenya. Women in our study demonstrated preferences for both long‐ and short‐acting PrEP modalities and different reasons for these preferences, indicating the need for client‐centred PrEP programmes that offer diverse choices for HIV prevention and empower women to make the choice best suited to their needs and lifestyle. Some of these preferences, such as safety for maternal–infant dyad, were specific to the pregnant‐postpartum period, while others likely reflected general preferences of women about PrEP. Overall, further efforts are needed to increase the choice and accessibility of various PrEP options to benefit the wellbeing of pregnant and postpartum women and their infants.

## COMPETING INTERESTS

The PrEP‐PP study received the study drug (Truvada®) from Gilead Sciences (Foster City, CA, USA).

## AUTHORS’ CONTRIBUTIONS

The study was conceived by NJW and designed by NJW, RH, JP and DLJD. NJW oversaw study implementation and data collection, analyzed the data, wrote the first draft of the manuscript and reviewed the manuscript following revision by all co‐authors. RM, MMM, LG and NM reviewed the study design, oversaw study implementation and data collection, and assisted with data analysis. CO, CM, RH, JP and DLJD reviewed the study design, study data and data analysis. JK, GJ‐S and JP designed the PrIMA‐X study, and LM and DLJD designed the PrEP‐PP study. All authors contributed to the development of this manuscript, and have read and approved the final manuscript.

## FUNDING

This work was funded by the National Institute of Mental Health: R01MH116771 (DJD, LM), The Eunice Kennedy Shriver National Institute of Child Health and Human Development: 1R01HD106821 (DJD) R01HD100201 (JP), the Fogarty International Center: K01TW011187 (DJD), the National Institute of Nursing Research: R01NR019220 (JP and JK) and the National Institutes of Allergy and Infectious Diseases: R01AI125498 (GJS).

## Supporting information


**Table S1**: Descriptions provided to study participants of long‐acting PrEP modalities in development/undergoing approval process.
**Table S2**: Reasons behind preference of long‐acting PrEP methods versus oral PrEP methods by pregnant and postpartum women.

## Data Availability

The data that support the findings of this study are available upon request from the corresponding author, nwara@mednet.ucla.edu.
