# Supplementary material for: Preferences and acceptability for long‐acting PrEP agents among pregnant and postpartum women with experience using daily oral PrEP in South Africa and Kenya
Source: J Int AIDS Soc. 2023 May 23;26(5):e26088. doi: 10.1002/jia2.26088 (PMC10206415; doi:10.1002/jia2.26088)
Supplement: Supplementary file 1 — Table S1: Descriptions provided to study participants of long‐acting PrEP modalities in development/undergoing approval process. Table S2: Reasons behind preference of long‐acting PrEP methods versus oral PrEP methods by pregnant and postpartum women. [file JIA2-26-e26088-s001.docx]

**Supplementary Table 1: Descriptions provided to study participants of long-acting PrEP modalities in development/undergoing approval process**

| **CAB-LA description[19]:** | Research is ongoing to develop an injectable version of PrEP that can protect against HIV. This is an injection that is administered once every 8 weeks. Efficacy data shows that the injection reduces HIV risk by almost 90% compared to oral PrEP. The most common side effects were pain, redness and swelling at the site of the injection, but very few participants dropped out of the studies due to these reactions. Like oral PrEP, the injection does not protect against other STIs or unwanted pregnancy. Studies are still ongoing currently to understand the safety of providing an HIV prevention injection to pregnant women seeking to prevent HIV. |
| --- | --- |
| **Vaginal ring PrEP description[22]:** | Research is ongoing to develop a vaginal ring that can protect against HIV. This is a flexible, silicone ring that is inserted into the vagina and is effective for a full month at a time. Efficacy data shows that the ring reduces HIV risk by about 30-35%. Because the ring delivers the drug directly to the vagina, side effects may be reduced. Many women in existing studies reported forgetting the ring was in place, and that neither they nor their partner could feel it during sex. Like oral PrEP, the ring does not protect against other STIs or unwanted pregnancy. Studies are still ongoing currently to understand the safety of providing the ring to pregnant women seeking to prevent HIV. |

**Supplementary Table 2: Reasons behind preference of long-acting PrEP methods versus oral PrEP methods by pregnant and postpartum women**

| **If injectable PrEP was approved as safe for pregnant women to use, would you prefer to keep using oral PrEP or switch to the injection?** | **Overall (N=394)** | **South Africa (n=190)** | **Kenya (n=204)** | **p-value** |  |
| --- | --- | --- | --- | --- | --- |
| Keep using oral PrEP | 94 (24) | 48 (25) | 46 (23) | 0.53 |  |
| Switch to injectable PrEP | 297 (75) | 141 (74) | 156 (76) | 0.60 |  |
| Unsure | 3 (0.8) | 1 (0.5) | 2 (1) | 0.53 |  |
| For those who selected injectable PrEP over oral PrEP: What characteristics about the injection make you interested in switching?* |  | | | ***p*-value** |  |
|  | **Overall (n=297)** | **South Africa (n=141)** | **Kenya (n=156)** |  |  |
| Longer duration/once every 8 weeks | 188 (63) | 123 (87) | 65 (42) | <0.01 |  |
| Not having to remember to take a pill every day | 144 (48) | 80 (57) | 64 (41) | <0.01 |  |
| Easy to keep private from my partner/others | 83 (28) | 7 (5) | 76 (49) | <0.01 |  |
| Administered by medical professional | 40 (13) | 9 (6) | 31 (20) | <0.01 |  |
| Not carrying pills | 83 (28) | 18 (13) | 65 (42) | <0.01 |  |
| Other (e.g., fewer potential side effects, comfort with contraceptive injection) | 5 (2) | 5 (4) | 0 (0) | 0.02 |  |
| For those who selected “Oral PrEP” or “Unsure”: What characteristics about the injection make you not interested or unsure about switching?* |  | | | ***p*-value** |  |
|  | **Overall (n=97)** | **South Africa (n=49)** | **Kenya (n=48)** |  |  |
| Injection pain | 71 (73) | 37 (76) | 34 (71) | 0.60 |  |
| Potential side effects | 24 (25) | 15 (31) | 9 (19) | 0.18 |  |
| Not enough safety data (i.e. afraid of impact on me) | 13 (13) | 2 (4) | 11 (23) | <0.01 |  |
| Worry about impact on baby's health after delivery due to being on PrEP while breastfeeding | 6 (6) | 0 (0) | 6 (13) | 0.01 |  |
| Worry about impact on having a healthy pregnancy due to PrEP | 3 (3) | 0 (0) | 3 (6) | 0.12 |  |
| Once every 8 weeks | 1 (1) | 0 (0) | 1 (2) | 0.50 |  |
| Other (e.g., needle fears, does not want more clinic, mistrust of contraceptive injection, used to oral PrEP) | 5 (5) | 5 (10) | 0 (0) | 0.03 |  |
| **If the vaginal ring was approved as safe for pregnant or breastfeeding women to use, would you prefer to keep using oral PrEP or switch to the vaginal ring?** | **Overall (N=394)** | **South Africa (n=190)** | **Kenya (n=204)** | ***p*-value** |  |
| Keep using oral PrEP | 344 (87) | 165 (87) | 179 (88) | 0.79 |  |
| Switch to vaginal ring | 40 (10) | 22 (12) | 18 (9) | 0.37 |  |
| Unsure | 10 (3) | 3 (2) | 7 (3) | 0.20 |  |
| For those who selected vaginal ring over oral PrEP:  What characteristics about the ring make you interested in switching?* |  | | | ***p*-value** | |
|  | **Overall (n=40)** | **South Africa (n=22)** | **Kenya (n=18)** |  |  |
| Longer duration/once a month | 17 (43) | 14 (64) | 3 (17) | <0.01 | |
| Not having to remember to take a pill every day | 17 (43) | 12 (55) | 5 (28) | 0.09 | |
| Easy to use | 17 (43) | 9 (41) | 8 (44) | 0.82 | |
| Not carrying pills | 7 (18) | 1 (5) | 6 (33) | 0.02 | |
| Unable to feel it | 3 (8) | 0 (0) | 3 (17) | 0.08 | |
| Easy to keep private from my partner/others | 3 (8) | 0 (0) | 3 (17) | 0.08 | |
| Fewer side effects | 2 (5) | 2 (9) | 0 (0) | 1 | |
| Other (familiarity with contraceptive ring/internal condom) | 2 (5) | 2 (9) | 0 (0) | 0.30 | |

| For those who selected “Oral PrEP” or “Unsure”: What characteristics about the ring make you not interested or unsure about switching?* |  |  |  | | | **p-value** |
| --- | --- | --- | --- | --- | --- | --- |
|  | **Overall (n=354)** | | | **South Africa (n=168)** | **Kenya (n=186)** |  |
| Insertion into vagina | 228 (64) | | | 138 (82) | 90 (48) | <0.01 |
| Side effects | 97 (27) | | | 36 (21) | 61 (33) | 0.02 |
| Not enough safety data (i.e. afraid of impact on me) | 89 (25) | | | 39 (23) | 50 (27) | 0.43 |
| User or partner may feel it | 51 (14) | | | 13 (8) | 38 (20) | <0.01 |
| PrEP vaginal ring only reduces risk by 30% | 37 (10) | | | 24 (14) | 13 (7) | 0.03 |
| Worry about impact on having a healthy pregnancy due to PrEP | 25 (7) | | | 5 (3) | 20 (11) | <0.01 |
| Worry about impact on baby's health after delivery due to being on PrEP while breastfeeding | 28 (8) | | | 6 (4) | 22 (12) | <0.01 |
| Once a month | 15 (4) | | | 3 (2) | 12 (7) | 0.03 |
| Other (e.g. reduced risk/discomfort with incorrect usage, comfort with pills, fear that ring is scented, general discomfort/mistrust, ashamed) | 14 (4) | | | 11 (7) | 3 (2) | 0.02 |

**Participants were asked open-ended questions regarding oral PrEP likes/dislikes and interviewer selected the all responses endorsed by the participant.*
